# Randomized Controlled Lifestyle Intervention (LION) Study for Weight Loss and Maintenance in Adults With Obesity—Design and Methods

**DOI:** 10.3389/fnut.2020.586985

**Published:** 2020-11-10

**Authors:** Anna Reik, Christina Holzapfel

**Affiliations:** Institute for Nutritional Medicine, School of Medicine, University Hospital “Klinikum Rechts der Isar”, Technical University of Munich, Munich, Germany

**Keywords:** personalized nutrition, obesity, low carb, low fat, formula diet, mobile application (app), lifestyle, weight maintenance

## Abstract

**Introduction:** Due to the increasing prevalence of obesity, approaches for a more effective treatment especially in the long-term perspective are needed. However, studies on weight loss and maintenance show heterogeneous results with large inter-individual variations. Therefore, it is of interest to identify factors that contribute to inter-individual differences and predict the success of long-term weight management.

**Methods and Analysis:** The primary outcome of the Lifestyle Intervention (LION) Study is to evaluate the effect of two diets (low carb vs. low fat) and two digital counseling tools (newsletter vs. mobile application) on weight maintenance 12 months after weight loss. The identification of predictive factors (e.g., genetic, epigenetic, physiological, psychological) for the success of weight loss and maintenance is a secondary outcome. Men and women with a body mass index (BMI) between 30.0 and 39.9 kg/m^2^, aged 18–65 years, and without severe diseases are considered eligible. After phenotyping (e.g., anthropometry, resting metabolic rate, meal challenges, blood parameters) participants will follow a formula-based, low-calorie diet (LCD) for 8 weeks. In addition, the intake of 200 g raw or cooked non-starchy vegetables are allowed per day. Subsequently, 252 participants will be randomized into one of the four intervention groups (low carb/app, low carb/newsletter, low fat/app, low fat/newsletter) for the 12-month weight maintenance step. The study will be concluded after another 12 months of follow-up. Results should provide indications for successful weight management and give insights into the personalized treatment of obesity.

**Ethics and Dissemination:** This study has been granted ethical approval by the local Ethics Review Committee of the School of Medicine, Technical University of Munich (vote: 69/19 S).

**Trial Registration Number:** This study has been registered within ClinicalTrials.gov (NCT04023942) and the German Clinical Trials Register (DRKS00017819).

## Introduction

The World Health Organization indicates that overweight affects globally 39% of the adult population, whereas obesity affects 13% of adults (1, 2). In Germany, the prevalence of overweight reaches 67.1% in men and 53.0% in women, whereas obesity reaches 18.9 and 22.5% of men and women, respectively (3).

Both overweight and obesity cause pathophysiological changes in the human body, increasing the risk for comorbidities, such as diabetes, cardiovascular diseases, or some cancers (1, 2, 4). Lifestyle based interventions for weight loss are well-established and are widely recommended in guidelines for the prevention and treatment of overweight and obesity (5).

Lifestyle interventions are usually centered around a “one-size-fits-all” strategy, being successful for the short-term (6). However, weight loss largely differs between individuals, and data on long-term weight maintenance have shown that most people regain weight after weight loss (5, 7). Studies have shown that mean weight loss is moderate with large inter-individual differences, ranging from 25 kg weight loss to 5 kg weight gain (8, 9), depending on intervention type and duration. Aspects that may affect inter-individual differences in weight regulation include lifestyle factors, environment, genetics, phenotype, metabolism, and the microbiome (5, 10, 11). There is still limited knowledge on which factors are associated with weight maintenance, and which therapeutic concepts are successful for weight maintenance. Taking this into consideration, innovative therapeutic approaches and guidance of affected people after weight loss are favorable to achieve weight changes in the long term. Therefore, there is an urgent need to identify predictors and concepts for long-term weight maintenance.

Personalization and digitalization of medical treatment are promising trends in the health care system. Although there are several definitions for “personalized nutrition,” personalization itself should incorporate aspects such as individual preferences, requirements, and habits, combined with key characteristics of an individual's phenotype and genotype to obtain an optimally tailored intervention strategy (6, 11). Which determinants can improve the effectiveness of personalized nutrition is current object of research.

Most research in this field is focused on gene-lifestyle interactions. The DIETFITS trial characterized people with obesity based on their genotypes and post-prandial insulin response. The participants were subsequently matched or mismatched to their “optimal” diet, based on their characteristics at baseline. However, no genotype-diet nor diet-insulin interaction was found for weight loss (8). On the other hand, the POUNDS Lost trial, as well as the DIRECT trial, have found that a specific diet can modify the expression of genes related to weight regulation, supporting weight loss and improving glucose metabolism (12). There is further evidence that the presence of specific genotypes might favor a greater reduction in weight and waist circumference (13).

Furthermore, the group of Zeevi et al. was able to integrate sociodemographic and microbiological characteristics to accurately predict the glucose response of an individual to a meal (10). Results of the POUNDS Lost trial reinforces the hypothesis that the gut microbiota plays a role in weight maintenance since it seems to be able to alter parameters of glucose metabolism (14). Fasting plasma glucose seems to be another determinant that should be considered when matching a diet to an individual's characteristics (4), making it a predictor for weight loss maintenance. However, the research around these types of biomarkers is still in the beginnings and has failed to show clinically relevant results (4, 5).

Several trials compared diets for the treatment of overweight and obesity. It is well-known, that a negative energy balance is relevant for weight loss (15). However, it has been shown that the macronutrient composition of a diet does not affect long term weight loss (9, 16). Low carb concepts lead to a greater initial weight loss compared to other dietary regimes, but have no advantages in the long-term. Other studies found out that low carb diets lead to beneficial metabolic changes (17, 18). Overall, it is rather unclear how low carb and low fat concepts compare regarding weight maintenance after weight loss. Therefore, low carb and low fat diet concepts are innovative in the context of weight loss maintenance and are not yet finally proven.

Finally, novel information and communication technologies (ICT) like wearables and mobile applications (apps) with individualized recommendations, self-monitoring features, or feedback systems may be a useful and inexpensive way to improve weight loss maintenance (5, 19). While self-weighing is known to be effective for weight maintenance, little is known as to whether self-monitoring technologies for tracking lifestyle helps people to maintain their weight. New ICTs promise to facilitate individualized communication at a low-cost level (20). Theses digital tools are not yet proven to support weight maintenance after weight loss.

Overall, weight loss maintenance is difficult for most people and there is a lack of evidence to support the hypothesis that a specific macronutrient composition of a diet or that digital tools can improve weight maintenance. Moreover, predictors for personalized dietary recommendations for weight loss and maintenance are still lacking. In this context, the Lifestyle Intervention (LION) Study aims to compare two different diets (low carb vs. low fat) and two digital tools (newsletter vs. app) for long term weight maintenance. Furthermore, genetic, epigenetic, physiological, psychological, and lifestyle predictors for successful weight loss and weight maintenance should be identified. The overall aim is to contribute to the development of a personalized treatment of obesity. A therapeutic approach tailored to the individual and adapted to their genetic background and metabolic conditions may result in greater weight loss success and maintenance in the long-term.

## Methods and Analysis

### Study Design

**Figure 1:**
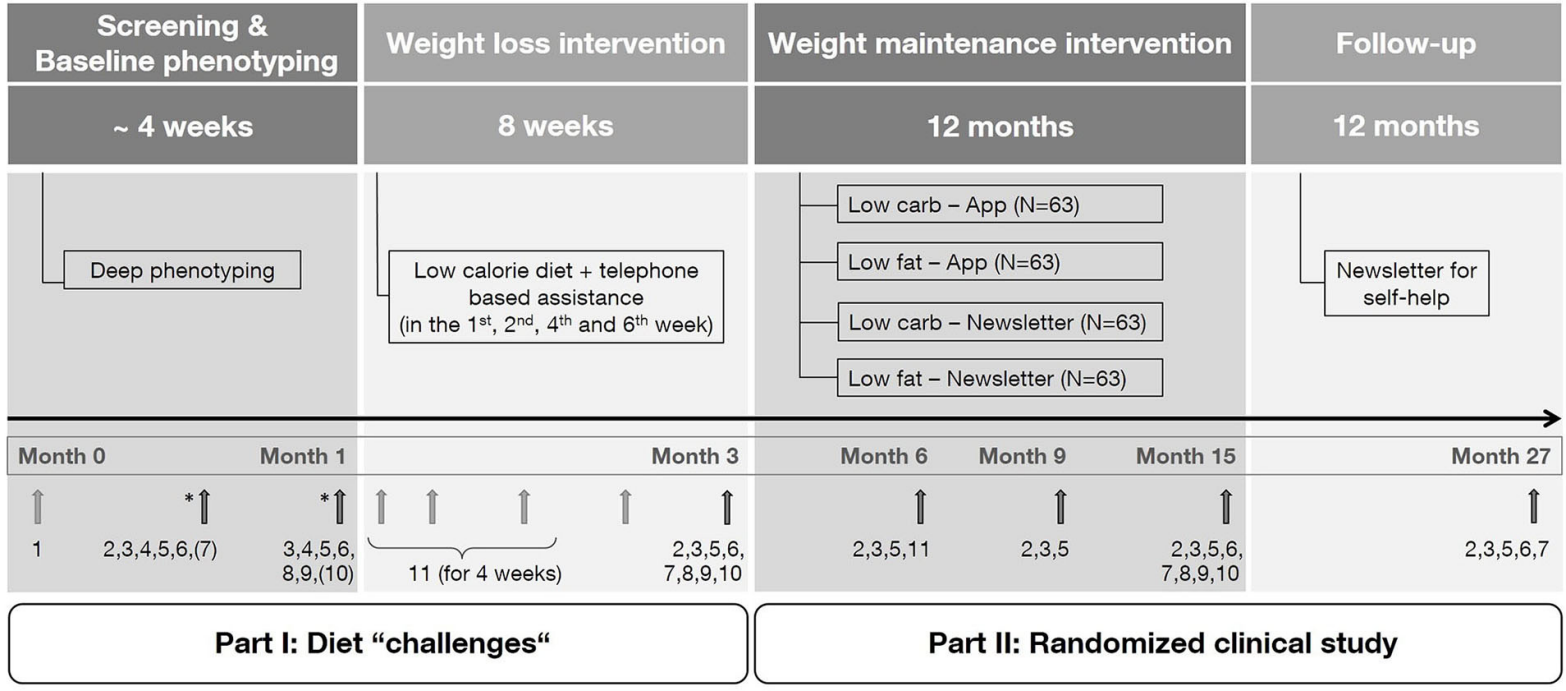
Study design. Numbers indicate measurements performed during the corresponding visit. (1) Screening questionnaire, (2) anthropometry, (3) collection of biomaterial, (4) meal challenge, (5) questionnaires/protocols, (6) vital parameters, (7) resting metabolic rate, (8) magnetic resonance imaging (subgroup), (9) physical functional performance test (subgroup), (10) hand strength measurement, (11) continuous glucose measurement. * Randomization of the test meal and selected measures. The exact time point of measurements marked by a bracket is dependent of randomization.

The LION Study is a 2 × 2 factorial randomized controlled trial in adults with obesity and comprises four steps: screening and baseline phenotyping (step I), a 8-week weight loss intervention (step II), a 12-month weight maintenance intervention (step III), and a 12-month follow-up period (step IV) (Figure 1). Measurements and data collection occur during face-to-face visits at different time points during the study. However, in individual cases (e.g., due to coronavirus pandemic) face-to-face visits can be converted to phone visits, in which self-reported data will be collected instead of measured data (21). Participants will be enrolled for 27 months from screening to final follow-up and will attend seven visits in total: two visits at baseline (month 1) and one visit each at 3, 6, 9, 15, and 27 months.

### Study Setting

The LION Study is a single-site study and takes place at the Human Study Center of the Institute for Nutritional Medicine, School of Medicine, Technical University of Munich, Germany. The first patient was enrolled in July 2019. Date for last patient out is predicted to be not before spring of 2023.

### Eligibility Criteria

Adults aged between 18 and 65 years, with a body mass index (BMI) between 30.0 and 39.9 kg/m^2^, in ownership of a smartphone and without any severe health condition, that may pose risk for the participant and affect the validity of our findings, are considered eligible. Table 1 specifies all inclusion and exclusion criteria. The participants' eligibility is self-reported and is assessed during a preliminary telephone-based screening interview. In exceptional cases, eligibility is assessed based on the judgment of the investigator on a by-case decision. Individuals who meet inclusion criteria (Table 1) are scheduled for a face-to-face visit.

**Table 1:** Main inclusion and exclusion criteria (self-reported, unless otherwise indicated).

Inclusion criteria	• Age: 18–65 years • Body mass index (BMI): 30.0–39.9 kg/m^2^ (at the scale of the study center) • Ownership of a smartphone with an internet connection (checked by the study team) • No active severe disease • Written informed consent
Exclusion criteria^*^	a. Medical conditions • Diabetes mellitus (Fasting plasma glucose >126 mg/dl, determined either by a rapid tester and laboratory findings) • Anemia (Hemoglobin <12 g/dl, determined either by a rapid tester and laboratory findings) • Severe cardiovascular (e.g., angina, myocardial infarction, stroke), liver (e.g., hepatitis B or hepatitis C), kidney and/or respiratory disease • Clinically significant untreated hypertension (measured and evaluated by the study physician) • Neoplasms (e.g., cancer or gastric ulcer) • Inflammatory bowel disease (e.g., Crohn's disease, ulcerative colitis) • Severe infections/inflammations (chronic or at time of recruitment) • Severe mental, behavioral or neurodevelopmental disorders (e.g., eating disorders, severe depression) • Severe diseases of the nervous system (e.g., Multiple sclerosis, Parkinson‘s disease, epilepsy) • Untreated endocrine diseases (e.g., thyroid disease, determined by laboratory findings) • Severe food allergies or intolerances, that are clinically significant • Severe Lipedema, that is clinically significant b. Medication Medications with the potential of affecting weight, energy expenditure, or the participant's judgment ability (such as glucocorticoids, psychoactive medication, or epileptic medication) are not allowed. The decision will take place on a by-case basis after evaluation of type, dose and usage of the medication by the study's physician. c. Other • Pregnancy and lactation (self-reported or determined by a pregnancy test) • Vigorous weight fluctuations (>5 kg) in the last 3 months • Immobility • Surgery in the last 3 months and bariatric surgery • Participation in other intervention studies, which could influence the study outcomes • Carrier of pacemakers • Blood donation or transfusion in the last 3 months

* *In general, a case and/or condition not named specifically is not an exclusion criterion for enrollment in the first instance. However, if a criterion is considered critical in the judgment of the investigator, the participant is excluded. Additionally, a belated inclusion/exclusion is possible depending on the present case. The study physician makes a final decision on a by-case basis, after estimation of the clinical significance*.

Participants undergo clinical examinations during the first two face-to-face visits (Visit 1A and 1B), serving primarily for baseline phenotyping as well as for the detection of potentially unknown exclusion criteria. For preliminary screening, a drop of capillary blood is withdrawn and fasting plasma glucose, as well as hemoglobin levels, are directly measured at the study center to exclude participants with undiagnosed diabetes mellitus (impaired glucose tolerance is accepted) and/or anemia beforehand. Table 1 specifies the threshold values for glucose and hemoglobin. Fasting glucose and hemoglobin levels of capillary blood are analyzed with the rapid tester HemoCue® Glucose 201+ System (HemoCue AB, Sweden) and DiaSpect Tm Analyser (EKF Diagnostic GmbH, Germany), respectively.

### Interventions

#### Weight Loss Intervention

Once the participant is still deemed eligible after baseline phenotyping (step I), the 8-week formula-based low-calorie diet (LCD) starts as weight loss intervention. Formula products (Itrim, Itrim Sverige AB, Sweden) are provided for the participants free of charge and are regularly handed out at pick up appointments. Each meal provides 200 kcal. A total daily calorie intake of 800 kcal exclusively by formula products is predetermined, resulting in four formula products per day. To increase compliance in the weight loss step, an additional daily intake of 200 g raw or cooked non-starchy vegetables is allowed. Due to further consumption of vegetables, the daily calorie intake is higher than 800 kcal, whereby the weight loss step is classified as LCD. During the LCD, participants fill out a food diary to record consumed products including calorie-free beverages (e.g., water, tea, coffee, beverages sweetened with artificial sweeteners) and vegetables and to document well-being or side effects. Furthermore, participants are phenotyped by a continuous blood glucose monitor (FreeStyle Libre Pro, Abbott Diabetes Care Inc., USA) during the first 4 weeks of intervention.

During the weight-loss period, participants are contacted regularly by phone (at weeks 1, 2, 4, and 6) to ensure well-being, to record observed side effects and to increase compliance. After 8 weeks of LCD, participants attend a face-to-face visit (Visit 2) at the study center, which ensures a seamless transition to the weight maintenance step.

#### Weight Maintenance Intervention

In order to exclude participants with either no or low adherence to the formula diet, only participants that achieved a weight loss over 4 kg after 8 weeks of LCD, are eligible for randomization into one of the four weight maintenance intervention arms: (a) low carb and app-based group, (b) low carb and newsletter-based group, (c) low fat and app-based group, (d) low fat and newsletter-based group.

Low carb is defined as 30 energy percent from carbohydrates, 50 energy percent from fat, and 20 energy percent from protein. Low fat is defined as 25 energy percent from fat, 55 energy percent from carbohydrates, and 20 energy percent from protein. This nutrient composition was firstly defined to allow the protein intake of both groups to be standardized and comparable, and secondly, to ensure feasibility and compliance to the weight maintenance diet for the long-term. There is no further breakdown specifying fat or carbohydrate types in the weight maintenance diet. However, participants are trained to make healthier decisions with the help of an overview which foods should be avoided and which better alternatives are available (providing information on e.g., low carb/low fat preparation of food, choosing low energy but nutrient-dense alternatives, or choosing foods with lower fat/lower carb content in the same food group).

The daily energy requirement is calculated for each participant individually, based on the measured resting metabolic rate (RMR) after weight loss multiplied with the individual's physical activity level (PAL). The PAL is chosen based on the PAL values for work and leisure activities of the German Nutrition Society, according to self-reported work, leisure, and physical activities. However, the recommended daily energy intake in the weight maintenance period is 10% lower than the calculated daily energy requirement (recommended daily energy intake = (RMR x PAL) – 10%). The reduction by 10% serves as a safety buffer to ensure weight maintenance, to diminish known inaccuracies in the calculation of energy requirements. If desired, further weight loss is allowed during the weight maintenance step, provided that it is performed within the framework of the weight maintenance intervention. The participant's compliance is estimated based on the recurring determination of blood and urine parameters (e.g., urea concentration for protein intake), and macronutrient composition of total energy intake assessed by a dietary record at three time points during the 12-month weight maintenance step. At months 6, 9, and 15 participants are examined in face-to-face visits (Visit 3A, 3B, and 3C).

Participants assigned to the app-based intervention group, regularly have contact with a coach via the app (Oviva, Oviva AG, Switzerland), providing nutritional guidance and support during the 12-month weight maintenance step. In the beginning, there is a single phone call between coach and participant to get to know each other and to become familiar with the participant's “starting point.” Afterward, conversations between coach and participant take place within the app's private chatroom in a decreasing frequency (weekly in the first 3 months, every 2 weeks in the following 6 months and once a month in the last 3 months of intervention). Additionally, participants are provided with a monthly information sheet (LION-Info) containing information (e.g., recipes, shopping lists, preparation of food) on the assigned diet and tips for long-term weight maintenance. Content to be discussed between coach and participant is standardized by the study team. However, the participant can address specific dietary questions to the coach. All coaches are certified nutritionists and are additionally trained by the study team. Tracking of weight/physical activity and uploading pictures of meals (as a picture-based dietary record) are some examples of further features within the app, which are not mandatory for the intervention but can be additionally used by the participants.

In comparison, participants assigned to the newsletter-based intervention group get digital newsletters regularly via email, providing nutritional information (e.g., recipes, shopping lists, preparation of food, basic nutritional facts, quizzes) regarding the assigned diet in the same frequency as “contacts” take place in the app-based group. Information included in the LION-Info is also provided in the newsletter. However, the newsletter is kept more general, to be appealing to a broader group of people. Content to be discussed in the newsletter is standardized by the study team and is identical to the LION-Info sent to the app-based intervention group.

#### Follow-Up

During the 12-month follow-up, all participants receive a newsletter with general information on self-help for long-term weight maintenance every 3 months, independent of the assigned weight maintenance intervention group. Topics to be mentioned include not only nutritional information, but also refer to psychological aspects and physical activity for weight maintenance. After 12 months, the final face-to-face visit (Visit 4) takes place, concluding the study participation.

### Meal Challenges

**Figure 2:**
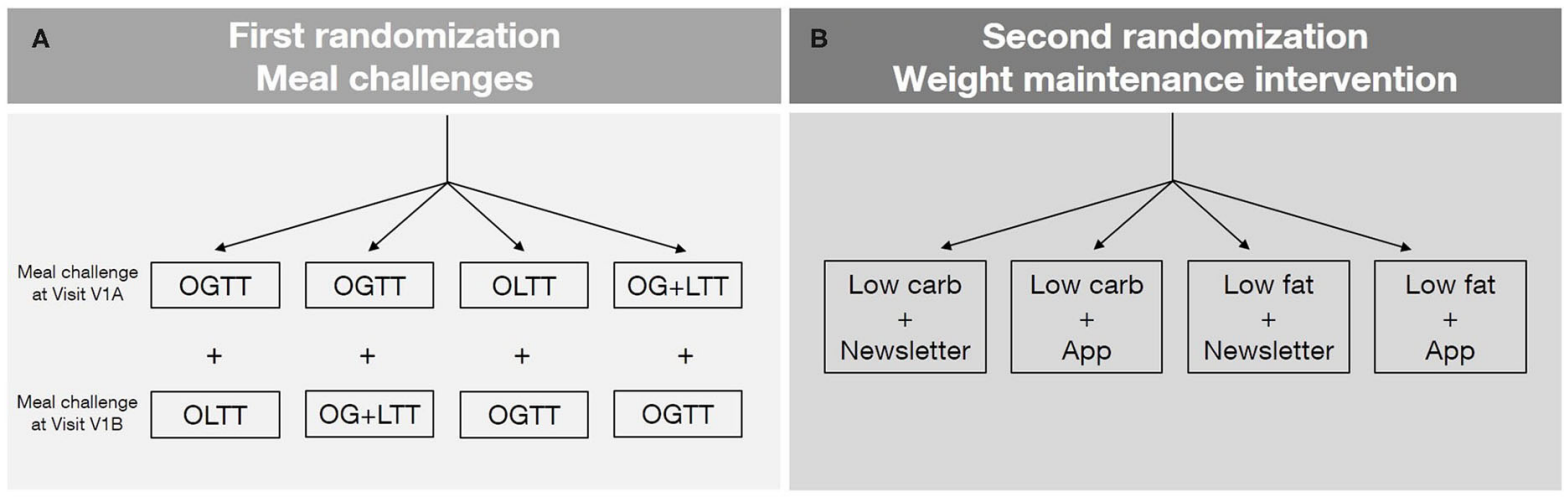
**(A)** First randomization: meal challenges. After the telephone-based screening and before the first visit, the order of meal challenges as well as the type of lipid meal challenge will be randomized. Two meal challenges are carried out per participant (one during Visit V1A and one during Visit V1B). Oral glucose tolerance test (OGTT), oral glucose and lipid mixed meal challenge (OG+LTT), oral lipid tolerance test (OLTT). **(B)** Second randomization: weight maintenance intervention. After weight loss (during the Visit V2), participants are randomized to one of the four intervention groups. The second randomization is stratified according to the parameters of the first randomization. No results of the meal challenge tests (blood and other metabolic parameters) are considered for randomization.

For phenotyping purposes and analysis of individual metabolic responses as a secondary outcome, participants take part in two meal challenges at baseline (Visits V1A and V1B). All participants undergo an oral glucose tolerance test (OGTT) and a lipid meal challenge, which itself can be based on a plain lipid drink (oral lipid tolerance test, OLTT) or a glucose-lipid mixed meal challenge (oral glucose and lipid tolerance test, OG+LTT) (Figure 2). Blood is drawn at baseline and several time points after administration of the test drink, to monitor changes of metabolites in the blood (e.g., glucose, insulin, and lipid profile) (**Table 3**).

The OGTT is carried out after an overnight fast after consumption of a 300 ml drink providing 75 g glucose. The OGTT-drink is prepared as follows: 82.5 g glucose monohydrate (≥99.5% D(+)-glucose monohydrate, Ph. Eur.; Carl Roth GmbH + Co. KG, Germany) filled up to 300 ml with tepid water. Blood is drawn at baseline and 30, 60, 90, and 120 min after drinking the test meal.

The lipid meal challenge is also carried out after an overnight fast. The type of the administered lipid meal challenge (plain lipid drink or a glucose-lipid mixed drink) is randomly chosen for each participant. The glucose-lipid mixed meal challenge (OG+LTT) is applied as an attempt to modulate different processes of metabolic health in comparison to a plain glucose or plain lipid meal challenge. The OLTT-drink is prepared as follows: a fat emulsion drink (Calogen®, Nutricia GmbH, Germany) is filled up with water to 300 ml. The fat emulsion drink provides 35 g fat per m^2^ of the participant's body surface, which is calculated based on the equation by Du Bois and Du Bois (22). The 300 ml OG+LTT-drink is prepared analogous to the plain lipid drink and contains additionally 82.5 g glucose monohydrate. Blood is drawn at baseline and 30, 60, 120, 180, 240, and 300 min after drinking the test meal.

To guarantee a washout period, without distorting the baseline data, the two first visits (Visit 1A and 1B) should be at least 3 days apart.

### Outcomes

The primary objective of the LION Study is to evaluate the effect of two diets (low carb vs. low fat) and two digital counseling tools (app vs. newsletter) on long-term weight maintenance 12 months after weight loss (weight change from baseline to month 15, Visit 3C).

Secondary objectives are mainly focused on the identification of predictors for the success of weight loss and weight loss maintenance. This includes data such as anthropometric, metabolic, and genetic parameters; RMR; clinical, lifestyle, psychological, environmental, and social factors; adipose tissue and fat compartments measured by magnetic resonance imaging (MRI, subgroup); among others. The characterization of metabolic responses on meal challenges at baseline (Visits V1A and V1B; Tables 2, 3) that may predict the success of weight loss and weight loss maintenance is one of the main secondary objectives.

**Table 2:** Data collection plan.

	**Time point**							
**Study step**	**Screening and baseline phenotyping**			**Weight loss**	**Weight maintenance**			**Follow-up**
**Visit**	**Screening**	**V1A**	**V1B**	**V2**	**V3A**	**V3B**	**V3C**	**V4**
**Parameters**								
Screening questionnaire	x							
Verify eligibility	x	x	x					
Informed consent	x	x						
Anthropometry		x		x	x	x	x	x
Body height		x						
Bodyweight		x		x	x	x	x	x
Body composition^a^		x		x	x	x	x	x
Body circumferences^b^		x		x	x	x	x	x
Vital parameters		x	x	x			x	x
SBP, DBP		x	x	x			x	x
Heart rate		x	x	x			x	x
Body temperature		(x)^*^	(x)^*^	x			x	x
Respiratory rate		(x)^*^	(x)^*^	x			x	x
Biomaterials		x	x	x	x	x	x	x
Blood		x	x	x	x	x	x	x
Urine			x	x	x		x	
Saliva			x	x			x	
DBS		x		x			x	
24-h urine (optional)			x	x	x		x	
Stool (optional)			x	x			x	
PBMCs (subgroup)		x	x	x			x	
Metabolic response								
Glucose tolerance test		(x)^*^	(x)^*^					
Lipid tolerance test		(x)^*^	(x)^*^					
Questionnaires		x	x	x	x	x	x	x
BAS			x	x	x	x	x	x
DEBQ			x	x	x	x	x	x
EDE-Q8		x						
IWQOL-lite			x	x	x	x	x	x
MBSRQ-AS			x	x	x	x	x	x
mYFAS 2.0			x	x	x	x	x	x
PHQ-9			x				x	
Nutritional assessment		x		x	x	x	x	x
FFQ		x						x
Dietary diary				x				
Dietary record					x	x	x	
PA assessment		x	x	x	x	x	x	x
MoMo		x		x	x	x	x	x
7-day step log			x	x	x	x	x	
RMR		(x)^*^	(x)^*^	x			x	x
Hand strength		(x)^*^	(x)^*^	x			x	
CGM				x				
Functional tests (optional)			x	x			x	
MRI (optional)			x	x			x	
Safety record				x	x	x	x	

*BAS, body appreciation scale; CGM, continuous glucose monitor; DBS, dried blood spots; DBP, diastolic blood pressure; DEBQ, dutch eating behavior questionnaire; EDE-Q8, eating disorder examination questionnaire; FFQ, food frequency questionnaire; IWQOL-lite, impact of weight on quality of life-lite questionnaire; MBSRQ-AS, multidimensional body-self relations questionnaire—appearance scales; MoMo, motoric-module questionnaire; MRI, magnetic resonance imaging; mYFAS 2.0, modified yale food addiction scale version 2.0; PA, physical activity; PBMCs, peripheral blood mononuclear cells; PHQ-9, patient health questionnaire—depression module; RMR, resting metabolic rate; SBP, systolic blood pressure*.

* *Order of measurements dependent on randomization*.

a *Body composition: fat mass, fat-free mass, total body water, impedance*.

b *Waist circumference, hip circumference*.

**Table 3:** Routine blood and urine parameters.

	**Time point**						
**Study step**	**Screening and baseline phenotyping**		**Weight loss**	**Weight maintenance**			**Follow-up**
**Visit**	**V1A**	**V1B**	**V2**	**V3A**	**V3B**	**V3C**	**V4**
**Blood parameters**							
CBC	x		x	x	x	x	x
AST	x		x	x	x	x	x
ALT	x		x	x	x	x	x
γ-GT	x		x	x	x	x	x
AP	x		x	x	x	x	x
Sodium	x		x	x	x	x	x
Potassium	x		x	x	x	x	x
Calcium	x		x	x	x	x	x
Albumin	x		x				
Creatinine	x		x	x	x	x	x
GFR	x		x	x	x	x	x
Urea	x		x	x	x	x	x
Uric acid	x		x	x	x	x	x
Total protein	x		x	x	x	x	x
Bilirubin	x		x				
CRP	x		x	x	x	x	x
HbA1c	x		x	x	x	x	x
TSH	x		x			x	x
T3	x		x			x	x
T4	x		x			x	x
Glucose	x^*^	x^*^	x	x	x	x	x
Total cholesterol	x^*^	x^*^	x	x	x	x	x
Triacylglycerol	x^*^	x^*^	x	x	x	x	x
HDL-C	x^*^	x^*^	x	x	x	x	x
LDL-C	x^*^	x^*^	x	x	x	x	x
LDL/HDL ratio	x^*^	x^*^	x	x	x	x	x
Insulin	x^*^	x^*^	x	x	x	x	x
**Urine parameters**							
Albumin		x	x				
Creatinine		x	x	x		x	
Urea		x	x	x		x	
Urea nitrogen		x	x	x		x	

*ALT, alanine aminotransferase; AP, alkaline phosphatase; AST, aspartate aminotransferase; CBC, complete blood count; CRP, C-reactive protein; γ-GT, gamma-glutamyltransferase; GFR, glomerular filtration rate; GOT, glutamic oxalacetic transaminase; GPT, glutamic pyruvate transaminase; HbA1c, hemoglobin A1c; HDL-C, high-density lipoprotein cholesterol; LDL-C, low-density lipoprotein cholesterol; TSH, thyroid-stimulating hormone; T4, thyroxine; T3, triiodothyronine*.

* *Several time points are available for these parameters, since they will be analyzed as part of the meal challenge*.

Further exploratory research questions are planned and will be clarified in the course of the study.

### Sample Size and Power Calculation

A total of 252 participants will be randomized to the weight maintenance step (*n* = 63 per arm). Assuming a 30% dropout, there will be 176 participants (*n* = 44 per arm) that provide primary endpoint data. A sample size of 176 participants is sufficient to detect a difference in weight loss maintenance of 1.7 kg between the two dietary intervention arms and the two digital tools with a power of 80%, a significance level of 0.05 and assuming a within-arm standard deviation of 4.0 kg.

To randomize 252 participants to the weight loss maintenance step, it is estimated that 504 participants are needed to be included in the weight loss step. This estimate is based upon the assumption that 30% of participants will drop out during weight loss and that 20% will not reach the weight loss of 4 kg, which is necessary for randomization to one of the four weight maintenance groups. These are conservative estimates for dropout and ineligibility. If these rates are lower, fewer people will need to be screened and included in the weight loss step.

### Randomization

There are two randomization time points (Figure 2). The first randomization takes place at baseline, shortly before the participant attends the first visit and determines either the order of the two meal challenges (OGTT and lipid meal challenge) as well as the type of the lipid meal challenge (plain lipid drink or a glucose-lipid mixed drink). The second randomization takes place after weight loss during visit V2, in which participants are randomly assigned to one of the four weight maintenance intervention arms. This randomization step is stratified according to the order and type of meal challenges assigned with the first randomization. The results of the meal challenge tests (e.g., glucose response) are not considered for the weight maintenance intervention.

For each randomization, an online tool of the Münchner Studienzentrum (MSZ) is used to generate the allocation sequences. This online tool uses pre-defined randomization lists, created block-wise. Due to the nature of the intervention, it is not possible to blind the participants as well as the study team.

### Data Collection

Measurements and data collection occur during seven face-to-face visits throughout the study. These visits are numbered according to the study step (I–IV). If there are more than one visit assigned to a study step, they are additionally labeled in alphabetical order (A-C). Participants attend the visits fasted for at least 8 h, without being physically active in the past 12 h and no smoking on the day of the visit. Table 2 gives an overview of data collected at different time points in the study.

#### Anthropometry

Height (cm) is measured in a standing position, without shoes and looking straight using a stadiometer (Seca 214, Seca GmbH & Co., KG, Germany).

Body composition is assessed in a fasted state using a body composition scale (BC-418MA, Tanita Europe B.V., Netherlands), based on the Bioimpedance analysis (BIA). The participant is asked to take off shoes and empty the bladder before measurement. Since the participant is allowed to be dressed in light clothing, one kilogram is deducted for the assessment. The following data outcomes are recorded: weight (kg), fat mass (%, kg), fat-free mass (kg), total body water (kg), and impedance (Ω).

Waist and hip circumferences (cm) are measured in a standing position using a non-stretch measuring tape with a hock for easier reading.

Furthermore, participants are invited to an optional MRI measurement to determine e.g., fat distribution, organ fat (e.g., liver fat content), and brown adipose tissue (e.g., proton density fat fraction).

Additionally, optional phenotyping concerning muscular strength, motor function and posture analysis are assessed through different techniques: Body Composition using an air displacement plethysmograph (BOD POD, Cosmed S.r.l., Italy) and a body composition analyzer (InBody 770, InBody Europe B.V., Germany), posture analysis using a 3D Body Scanner (VITUS 3D Body scanner, Vitronic, Germany) and the Diers scan (DIERS formetric 4D, DIERS International GmbH, Germany) and motor and muscle function using a 3D force plate (OPTIMA force plate, Advanced Mechanical Technology Inc., AMTI, USA), and the IsoMed Back Module (D. & R. Ferstl GmbH, Germany).

#### Vital Parameters

Systolic and diastolic blood pressure (mmHg) and heart rate (bpm) are carried out in a seating position using a blood pressure monitor (BM 60 or BM 70, BEURER GmbH, Germany; M400 Intelli IT, OMRON Medizintechnik Handelsgesellschaft mbH, Germany) with a cuff. Blood pressure is calculated as the mean value of 3 performed measurements on the arm with the higher blood pressure measured with a 1-min rest in between. Body temperature (°C) is measured with an in-ear infrared thermometer (Thermoscan IRT 4020, Braun GmbH, Germany). The respiratory rate measurement is carried out manually by counting the patient's breath rate per minute.

#### Biomaterials

Fasting venous blood, fasting midstream urine and 24 h urine samples are collected and sent to an external lab (SYNLAB Medizinisches Versorgungszentrum Labor München Zentrum GbR, Munich, Germany) for the analysis of routine parameters (Table 2). Furthermore, serum and plasma blood samples, peripheral blood mononuclear cells (PBMCs), dried blood spots (DBS) and urine samples are stored at −80°C until needed for further analysis. PBMCs however, will be gradually frozen at −80°C in a separate freezing container (Nalgene® Mr. Frosty® Cryo 1°C Freezing Container, Thermo Fisher Scientific Inc., USA) for the first 3 days and transferred to a liquid nitrogen tank after the third day. Sample collection, pre-analytic steps, labeling, storage, packing, and shipment of samples is performed according to standard operating procedures (SOPs).

Additionally, saliva samples are collected in a fasting state (Table 3). Participants spit about 1–2 ml directly into a tube with a screw cap. Saliva samples are stored at −80°C until analysis.

Participants are also invited to collect stool samples at home. Samples are collected in tubes with and without a DNA stabilizer. Samples will be stored at −80°C until analysis.

#### Questionnaires

German versions of the following questionnaires are applied once or several times throughout the study course: Body Appreciation Scale (BAS) (23), the Dutch Eating Behavior Questionnaire (DEBQ) (24), the short version of the Eating Disorder Examination Questionnaire (EDE-Q8) (25), impact of weight on quality of life-lite questionnaire (IWQOL-lite) (26), the Multidimensional Body-Self Relations Questionnaire—Appearance Scales (MBSRQ-AS) (27), Modified Yale Food Addiction Scale Version 2.0 (mYFAS 2.0) (28, 29), the Patient Health Questionnaire—Depression module (PHQ-9) (30, 31), and the Motorik-Modul (MoMo) questionnaire (32, 33). Additionally, questionnaires to assess medical history, social-economic factors (e.g., marital status, qualification, employment, smoking status), safety and an evaluation of the study program are filled out by participants.

#### Lifestyle Assessment

Dietary intake is assessed in each step of the LION Study using different methods. A food frequency questionnaire (FFQ) is used (Visits V1A and V4) to obtain an overview of the participant's diet history (34). A daily dietary record is filled in during the weight-loss intervention to assess the intake of formula diet products, vegetables, and beverages and to collect data on well-being. Furthermore, open dietary records for 3 consecutive days are applied three times during weight maintenance, to assess total energy intake and the participants' adherence to their assigned weight maintenance diet.

Moreover, participants are encouraged to use any tool of choice (e.g., wearables, apps) to track physical activity and document daily steps in a 7-day step log at five time points.

#### Resting Metabolic Rate (RMR)

The RMR is measured through indirect calorimetry (COSMED Quark RMR 2013010303, COSMED S.r.l., Italy) with a canopy hood. Based on the measured oxygen consumption (VO_2_) and carbon dioxide production (VCO_2_) and using the equation after Weir (35), the software calculates RMR (kcal/day). The 30-min measurement is carried out after an overnight fast in a lying position at rest.

#### Handgrip Strength

Handgrip strength (kg) is measured with a handgrip dynamometer based on a hydraulic system (JAMAR®, Performance Health Supply Inc., USA). The measurement takes place in a seated position with the elbow flexed at 90° and rested on an armrest. The participant is instructed to press as hard as possible for a short period. The measurement is repeated six times (three times each arm). The maximum isometric strength of the hand and forearm muscles is the highest value of the six measurements.

#### Continuous Glucose Monitoring (CGM)

For CGM participants wear a small sensor (FreeStyle Libre Pro, Abbott Diabetes Care Inc., USA) on the back of the upper arm, during the first 4 weeks of formula diet. The sensor consists of a thin, flexible filament (0.4 × 5 mm) which is inserted, activated, and replaced by the study team in the uppermost skin layer and automatically monitors blood glucose levels (mg/dl) in the interstitial fluid continuously. Collected data are used for exploratory analyses and has no impact on the weight loss or weight maintenance intervention. Diabetes status, as an important factor associated with overweight and obesity, will be monitored through routine blood parameters collected during face-to-face visits.

### Data Management

Data management, monitoring, and randomization are conducted in cooperation with the local coordinating center for clinical studies, the Münchner Studienzentrum (MSZ), which is a member of the nationwide network of clinical coordinating centers (KKS). Most data are completed on hard copy case report forms (CRFs) and are subsequently entered into an electronic database (MACRO 4, InferMed Ltd., UK) by the study team. Access rights and login criteria are defined. Monitoring is carried out by the MSZ according to a monitoring plan.

### Statistical Analysis

To investigate the primary outcome, a comparison of groups in the weight maintenance step (app groups combined vs. newsletter groups combined and low carb groups combined vs. low fat groups combined at month 15) will be conducted based upon a linear regression model. A further regression model assesses a potential interaction between the dietary and mode of delivery interventions. A complete-case analysis and analysis using multiple imputations will be performed. Participants are analyzed according to groups to which they are randomized (intent-to-treat, ITT).

The continuous secondary endpoints at different time points are compared between the dietary and the mode of delivery groups using analogous linear regression models in complete-case analyses.

Exploratory analyses are performed to assess parameters as potential predictors for effective weight loss and weight maintenance. Exploratory analysis can be restricted to subgroups based on a per-protocol analysis. For instance, to address the bias of self-reported data obtained due to the implementation of telephone-based visits during the coronavirus pandemic, the mode of visits will be considered in statistical analysis (e.g., by adjusting according to the mode of the visit). Moreover, interaction terms (e.g., gene-diet interactions) will be considered as exploratory analyses. Since further weight loss is allowed during weight maintenance, weight trajectories during this period will be also one element of subgroup analysis (e.g., weight loss, stable weight, weight regain).

Finally, missing data are a challenge in long-term clinical studies. Depending on the amount of acquired missing data, statistical analysis will be adapted (e.g., imputation).

### Participant and Public Involvement

Participants were not involved in the development of research questions, study design and dissemination of results. However, participants are involved in recruitment by distributing flyers or by word of mouth. Participants are regularly asked to assess the burden of the intervention through feedback questionnaires and verbally during visits.

## Ethics and Dissemination

This study is performed following the ethical principles of the current version of the Declaration of Helsinki. The study protocol has been approved by the local Ethics Review Committee of the School of Medicine, Technical University of Munich (vote: 69/19 S). The trial has been registered within clinicaltrials.gov (NCT04023942) and the German Clinical Trials Register (DRKS00017819). Any protocol modifications will be submitted for review by the research ethics committee.

Participants give oral consent before the telephone-based screening interview and sign the informed consent for the LION Study as well as for the storage of data and biospecimen before any procedure is conducted.

Together with the local coordinating center for clinical studies, a risk stratification has been performed indicating no increased risk for the participants compared to standard treatment. Participants will be closely monitored by the study team.

Route insurance is additionally provided for all participants.

Participants themselves are free to discontinue their participation at any time, without the obligation to state the reason for withdrawal. Reasons for withdrawal will be documented when nominated by participants. Participants can also be discontinued from the study, at the discretion of the investigator.

Personal data is two-fold encoded to maintain privacy and confidentiality. Participants receive copies of available routine test results (e.g., blood and urine parameters, RMR) after every visit. Results of this study will be submitted for publication in peer-reviewed journals and presented at conferences. Press releases for the wide public are planned after the study is completed.

## Discussion

The LION Study is a large clinical trial comparing lifestyle interventions on a large scale. These lifestyle interventions are well-known for weight loss purposes and are applied here to explore their effects on long-term weight maintenance in people with obesity. Furthermore, the dietary intervention is combined with digital tools, for which its efficacy on weight maintenance has to be shown. Moreover, several aspects of deep phenotyping of participants are incorporated, in order to identify predictors for successful weight loss and maintenance. This approach should pave the way toward personalized nutrition. We anticipate, that the methodological diversity of this long-term study will enable the derivation of personalized recommendations for weight loss and weight maintenance based on the phenotype and genotype of an individual.

The study design, including two randomization steps, a 2 × 2-factorial design, a weight loss and a weight maintenance period, and a follow-up investigation 24 months after weight loss, are strengths of the LION Study. This design ensures a comparison of baseline values and intervention effects at different time points. Compared to other studies the primary outcome of the LION Study is weight maintenance 12 months after weight loss, for which a thorough power calculation is provided. Due to trained staff, SOPs, study monitoring, and data management by the local coordinating center for clinical studies, high quality of data is ensured. The LION Study mainly fulfills the criteria of the European Expert Guidelines, mentioned in the OBEDIS Core Variables Project. These guidelines emphasize, that defining a core set of variables is key to incorporate in randomized, controlled clinical trials of obesity interventions (36).

However, as a result of the nature of the LION Study, some limitations appear. Due to the study environment, with frequent contacts between the study team and participants, weight loss and weight maintenance results might be different from “real-life” settings. Additionally, the weight maintenance diets are focused on the distribution of macronutrients, to maintain better feasibility and adherence to the diet. For this reason, there are no specifications on fat or carbohydrate types for a healthy balanced diet. Furthermore, study results are biased by the participants themselves, since people participating in such studies are usually highly motivated. Deep phenotyping is a major aspect of this study. However, the weight loss and weight maintenance intervention of this study is not based on obtained phenotyping data. Therefore, this study is not primarily intended as a proof of concept of personalized nutrition, but rather a multidimensional approach to identify an optimal intervention method for each phenotype. In line with other weight loss and weight maintenance trials, it is expected that more female than male participants will be recruited. Finally, data on sleep and stress are not collected.

The LION Study is a randomized clinical study addressing the complex nature of weight loss and weight maintenance. Results will provide a deeper insight into the metabolism underlying weight management. Through pooling of various datasets, many aspects of weight loss and weight maintenance are covered and will pave the way to tailored nutritional recommendations for obesity treatment. The long-term aim is to identify predictors for a personalized lifestyle therapy for overweight and obesity.

## Data Availability Statement

The original contributions presented in the study are included in the article/supplementary material, further inquiries can be directed to the corresponding author/s.

## Ethics Statement

The studies involving human participants were reviewed and approved by Ethics Review Committee of the School of Medicine, Technical University of Munich. The patients/participants provided their written informed consent to participate in this study.

## Author Contributions

AR and CH developed the study concept and design, contributed equally in writing, revising, and editing the final manuscript. AR drafted the initial manuscript. All authors gave final approval of the version to be published and meet the ICMJE criteria for authorship.

## Conflict of Interest

CH is a member of the scientific advisory board of 4 sigma GmbH (Oberhaching). The funding body has no role in the design, in the collection of data, in the preparation of this manuscript, and in the decision to submit the paper for publication. The remaining author declares that the research was conducted in the absence of any commercial or financial relationships that could be construed as a potential conflict of interest.

## References

[1] World Health Organization Global Status Report on Noncommunicable Diseases 2014. Geneva: World Health Organization (2014).

[2] World Health Organization Obesity and Overweight. Available online at: https://www.who.int/news-room/fact-sheets/detail/obesity-and-overweight (accessed January 07, 2020).

[3] Mensink GBM Schienkiewitz A Haftenberger M Lampert T Ziese T Scheidt-Nave C. Overweight obesity in Germany: results of the German health interview and examination survey for adults (DEGS1). Bundesgesundheitsblatt Gesundheitsforschung Gesundheitsschutz. (2013) 56:786–94. 10.1007/s00103-012-1656-3 23703499

[4] Hjorth MF Zohar Y Hill JO Astrup A. Personalized dietary management of overweight and obesity based on measures of insulin and glucose. Annu Rev Nutr. (2018) 38:245–72. 10.1146/annurev-nutr-082117-051606 29856931 PMC9105825

[5] Hall KD Kahan S. Maintenance of lost weight and long-term management of obesity. Med Clin North Am. (2018) 102:183–97. 10.1016/j.mcna.2017.08.012 29156185 PMC5764193

[6] Celis-Morales C Livingstone KM Marsaux CFM Macready AL Fallaize R O'Donovan CB. Effect of personalized nutrition on health-related behaviour change: evidence from the Food4Me European randomized controlled trial. Int J Epidemiol. (2016) 46:578–88. 10.1093/ije/dyw186 27524815

[7] Anderson JW Konz EC Frederich RC Wood CL Anderson JW Konz EC. Long-term weight-loss maintenance: a meta-analysis of US studies. Am J Clin Nutr. (2001) 74:579–84. 10.1093/ajcn/74.5.579 11684524

[8] Gardner CD Trepanowski JF Del Gobbo LC Hauser ME Rigdon J Ioannidis JPA. Effect of low-fat vs low-carbohydrate diet on 12-month weight loss in overweight adults and the association with genotype pattern or insulin secretion // effect of low-fat vs low-carbohydrate diet on 12-month weight loss in overweight adults and the association with genotype pattern or insulin secretion: the DIETFITS randomized clinical trial: the DIETFITS randomized clinical trial. J Am Med Assoc. (2018) 319:667–79. 10.1001/jama.2018.0245 29466592 PMC5839290

[9] Shai I Schwarzfuchs D Henkin Y Shahar DR Witkow S Greenberg I Weight loss with a low-carbohydrate, Mediterranean, or low-fat diet. N Engl J Med. (2008) 359:229–41. 10.1056/NEJMoa0708681 18635428

[10] Zeevi D Korem T Zmora N Israeli D Rothschild D Weinberger A. Personalized nutrition by prediction of glycemic responses. Cell. (2015) 163:1079–94. 10.1016/j.cell.2015.11.001 26590418

[11] Drabsch T Holzapfel CA. Scientific perspective of personalised gene-based dietary recommendations for weight management. Nutrients. (2019) 11:617. 10.3390/nu11030617 30875721 PMC6471589

[12] Huang T Wang T Heianza Y Sun D Ivey K Durst R. HNF1A variant, energy-reduced diets and insulin resistance improvement during weight loss: the POUNDS lost trial and DIRECT. Diabetes Obes Metab. (2018) 20:1445–52. 10.1111/dom.13250 29424957

[13] Celis-Morales C Marsaux CFM Livingstone KM Navas-Carretero S San-Cristobal R Fallaize R. Can genetic-based advice help you lose weight? Findings from the Food4Me European randomized controlled trial. Am J Clin Nutr. (2017) 105:1204–13. 10.3945/ajcn.116.145680 28381478

[14] Heianza Y Sun D Li X DiDonato JA Bray GA Sacks FM. Gut microbiota metabolites, amino acid metabolites and improvements in insulin sensitivity and glucose metabolism: the POUNDS lost trial. Gut. (2018) 68:263–70. 10.2337/db18-297-OR 29860242 PMC6275143

[15] Johnston BC Kanters S Bandayrel K Wu P Naji F Siemieniuk RA. Comparison of weight loss among named diet programs in overweight and obese adults: a meta-analysis. JAMA. (2014) 312:923–33. 10.1001/jama.2014.10397 25182101

[16] Sacks FM Bray GA Carey VJ Smith SR Ryan DH Anton SD. Comparison of weight-loss diets with different compositions of fat, protein, and carbohydrates. N Engl J Med. (2009) 360:859–73. 10.1056/NEJMoa0804748 19246357 PMC2763382

[17] Foster GD Wyatt HR Hill JO Makris AP Rosenbaum DL Brill C. Weight and metabolic outcomes after 2 years on a low-carbohydrate versus low-fat diet. Ann Intern Med. (2010) 153:147–57. 10.7326/0003-4819-153-3-201008030-00005 20679559 PMC2949959

[18] Foster GD Wyatt HR Hill JO McGuckin BG Brill C Mohammed BS. A randomized trial of a low-carbohydrate diet for obesity. N Engl J Med. (2003) 348:2082–90. 10.1056/NEJMoa022207 12761365

[19] Svetkey LP Stevens VJ Brantley PJ Appel LJ Hollis JF Loria CM. Comparison of strategies for sustaining weight loss: the weight loss maintenance randomized controlled trial. JAMA. (2008) 299:1139–48. 10.1001/jama.299.10.1139 18334689

[20] Thomas JG Bond DS Phelan S Hill JO Wing RR. Weight-loss maintenance for 10 years in the national weight control registry. Am J Prev Med. (2014) 46:17–23. 10.1016/j.amepre.2013.08.019 24355667

[21] European Commission. Guidance on the Management of Clinical Trials during the COVID-19 (Coronavirus) pandemic: Version 2 (27/03/2020). Available online at: https://ec.europa.eu/health/sites/health/files/files/eudralex/vol-10/guidanceclinicaltrials_covid19_en.pdf (accessed March 30, 2020).

[22] Du Bois D Du Bois EF. A formula to estimate the approximate surface area if height and weight be known 1916. Nutrition. (1989) 5:303–11. 2520314

[23] Swami V Stieger S Haubner T Voracek M. German translation psychometric evaluation of the body appreciation scale. Body Image. (2008) 5:122–7. 10.1016/j.bodyim.2007.10.002 18405871

[24] Nagl M Hilbert A de Zwaan M Braehler E Kersting A. The German version of the dutch eating behavior questionnaire: psychometric properties, measurement invariance, population-based norms. PLoS ONE. (2016) 11:e0162510. 10.1371/journal.pone.0162510 27656879 PMC5033316

[25] Hilbert A Tuschen-Caffier B Eating Disorder Examination-Questionnaire. Tübingen: Deutschsprachige Übersetzung.

[26] Kolotkin RL Crosby RD Kosloski KD Williams GR. Development of a brief measure to assess quality of life in obesity. Obes Res. (2001) 9:102–11. 10.1038/oby.2001.13 11316344

[27] Vossbeck-Elsebusch AN Waldorf M Legenbauer T Bauer A Cordes M Vocks S. German version of the multidimensional body-self relations questionnaire - appearance scales (MBSRQ-AS): confirmatory factor analysis and validation. Body Image. (2014) 11:191–200. 10.1016/j.bodyim.2014.02.002 24958652

[28] Meule A Muller A Gearhardt AN Blechert J. German version of the yale food addiction scale 2.0: prevalence and correlates of 'food addiction' in students and obese individuals. Appetite. (2017) 115:54–61. 10.1016/j.appet.2016.10.003 27717658

[29] Schulte EM Gearhardt AN. Development of the modified Yale food addiction scale version 2.0. Eur Eating Disord Rev. (2017) 25:302–8. 10.1002/erv.2515 28370722

[30] Löwe B Gräfe K Zipfel S Witte S Loerch B Herzog W. Diagnosing ICD-10 depressive episodes: superior criterion validity of the patient health questionnaire. Psychother Psychosom. (2004) 73:386–90. 10.1159/000080393 15479995

[31] Löwe B Spitzer RL Williams JBW Mussell M Schellberg D Kroenke K. Depression, anxiety and somatization in primary care: syndrome overlap and functional impairment. Gen Hosp Psychiatry. (2008) 30:191–9. 10.1016/j.genhosppsych.2008.01.001 18433651

[32] Schmidt S Will N Henn A Reimers A Woll A. Der Motorik-Modul Aktivitätsfragebogen MoMo-AFB. Karlsruhe: Leitfaden zur Anwendung und Auswertung (2016)

[33] Schmidt SCE Henn A Albrecht C Woll A Physical activity of german children and adolescents 2003-2012: the momo-study. Int J Environ Res Public Health. (2017) 14:1375 10.3390/ijerph14111375 PMC5708014 29137127

[34] Nöthlings U Hoffmann K Bergmann MM Boeing H Fitting portion sizes in a self-administered food frequency questionnaire. J Nutr. (2007) 137:2781–6. 10.1093/jn/137.12.2781 18029499

[35] Weir JB New methods for calculating metabolic rate with special reference to protein metabolism. J Physiol. (1949) 109:1–9. 10.1113/jphysiol.1949.sp004363 15394301 PMC1392602

[36] Alligier M Barrès R Blaak EE Boirie Y Bouwman J Brunault P OBEDIS core variables project: European expert guidelines on a minimal core set of variables to include in randomized, controlled clinical trials of obesity interventions. Obes Facts. (2020) 13:1–28. 10.1159/000505342 31945762 PMC7098277

